# Wound healing effects of *Asparagus lucidus* Lindl extract through the phosphorylation of ERK1/2

**DOI:** 10.1186/s12906-023-04066-w

**Published:** 2023-07-15

**Authors:** Minho Kim, Ki-Young Kim

**Affiliations:** 1 Graduate School of Biotechnology, Kyung Hee University, Seocheon, Giheung, Yongin-Si, Gyeonggi-do 446-701 Korea; 2 Department of Genetics and Biotechnology, College of Life Science, and Graduate School of Biotechnology, Kyung Hee University, Seocheon, Giheung, Yongin-si, Gyeonggi-do 446-701 Korea

**Keywords:** HaCaT cells, Proliferation, MAP kinase pathway, AP-1 transcription factor, VEGF

## Abstract

**Background:**

Skin is the outermost part of the human body and is essential in maintaining body homeostasis. In the event of skin injury, rapid wound repair is crucial to protect the body. In this study, we investigated the wound-healing properties of *Asparagus lucidus* Lindl extract by promoting keratinocyte proliferation.

**Methods:**

To evaluate the effect of *Asparagus lucidus* Lindl extract on skin regeneration, the 3-(4,5-dimethylthiazol-2-yl)-2,5-diphenyltetrazolium bromide assay was used to measure keratinocyte proliferation, while an in vitro wound-healing assay was performed to evaluate wound closure through keratinocyte re-epithelialization. The intracellular mechanisms of the extract were studied using Western blot analysis to measure the phosphorylated forms of mitogen-activated protein kinases and protein kinase B. The mRNA expression of cell cycle-related genes was analyzed using quantitative real time-PCR analysis. A murine in vivo wound-healing assay was also conducted to observe the effect of the extract on wound closure.

**Results:**

*Asparagus lucidus* Lindl extract induced 131.15% keratinocyte proliferation compared to the control in the 3-(4,5-dimethylthiazol-2-yl)-2,5-diphenyltetrazolium bromide assay. The in vitro wound-healing assay showed that the extract improved wound closure by 216.94% through keratinocyte re-epithelialization. Western blot analysis revealed that the phosphorylated form of extracellular signal-regulated kinase 1/2 was increased by extract treatment. Quantitative real time-PCR analysis showed a dose-dependent increase in the mRNA expression of *c-fos*, *c-jun*, and *VEGF*. The in vivo wound-healing assay showed a significant increase (22.13%) of wound closure compared to the control on day 5.

**Conclusion:**

*Asparagus lucidus* Lindl extract promotes keratinocyte proliferation by activating the extracellular signal-regulated kinase 1/2 pathway and up-regulating the mRNA expression of *c-fos*, *c-jun*, and vascular endothelial growth factor.

**Supplementary Information:**

The online version contains supplementary material available at 10.1186/s12906-023-04066-w.

## Introduction

The skin plays a crucial role in maintaining homeostasis of the human body. If the skin is injured, it is important to quickly heal in order to protect the body. The process of skin wound healing consists of four phases: inflammation, epithelialization, proliferation, and remodeling [1]. The proliferation of keratinocytes is crucial for wound healing because they are the main cell type of the epidermis. During the proliferation phase, basal epidermal keratinocytes proliferate and fill the wound through migration, maturation, and differentiation [2].

The mitogen-activated protein (MAP) kinase signaling pathway and phosphoinositide 3 kinase (PI3K)/protein kinase B (AKT) signaling pathway play a major role in the regulation of cell cycle progression [3]. Extracellular signal-regulated kinase (ERK), p38 mitogen-activated protein kinase (p38), and c-Jun N-terminal kinase (JNK) MAP kinases are activated by external wound-healing signals, such as cytokines and growth factors [4]. Activated ERK1/2 regulates the expression of cell cycle regulatory genes through cell cycle regulatory transcription factors like Activator Protein 1 (AP-1) [5–7]. Similarly, the PI3K/AKT pathway is induced by various signals including cell growth, proliferation, actin cytoskeleton organization, protein trafficking, transformation, and apoptosis [8, 9].

*Asparagus lucidus* Lindl (AL, Korean name is Cheonmun-dong, syn. *Asparagus cochinchinensis* (Lour.) Merr) is a perennial herbaceous plant from the lily family, and widely distributed in Northeast Asia. In Korea, AL inhabits the southern coastal areas and AL root is used as a traditional medicine. The physiological activity of AL extract has been reported for neuroprotection [10], anti-inflammation [11], anti-aging in the brain and liver [12], anti-oxidation [13], inhibition of apoptosis [14], and anti-bacterial activity [15]. AL extract also has benefits for the skin, such as soothing and reducing inflammation and irritation [16, 17].

However, the effects of AL extract on skin wound healing and the underlying mechanisms have not been studied. The aim of this study was to determine the wound healing properties of AL extract and the intracellular targets involved in keratinocyte proliferation in vitro and in vivo.

## Results

### AL extract induces keratinocyte proliferation

**Fig. 1:**
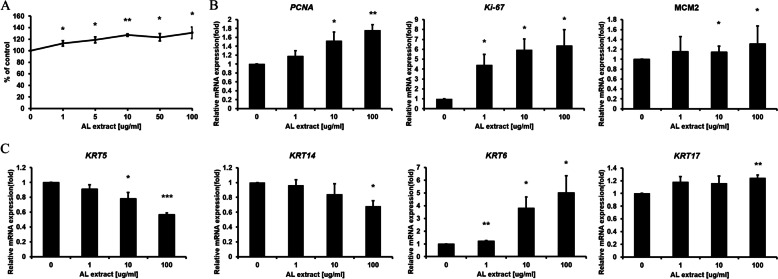
AL extract induces keratinocyte proliferation. **A** HaCaT cells were treated with the indicated concentration of AL extract to evaluate cell proliferation rate using the MTT assay. **B** Changes in mRNA expression of proliferation marker genes following treatment with AL extract. **C** Changes in mRNA expression of keratin genes following treatment with AL extract. (*p < 0.05, **p < 0.01, ***p < 0.001)

3-(4,5-dimethylthiazol-2-yl)-2,5-diphenyltetrazolium bromide (MTT) assay was performed on HaCaT cells to evaluate proliferation after treatment with AL extract at 0, 1, 5, 10, 50, and 100 μg/mL concentrations. Cell viability increased by 131.15% with 100 μg/mL AL extract treatment compared to the Dimethyl sulfoxide (DMSO) control (Fig. 1A).

Quantitative real time-PCR (qRT-PCR) was performed to examine changes in mRNA expression of proliferation markers (*PCNA*, *MCM2*, and *Ki-67*) and keratin genes (*KRT5/14/6/17*) [18]. The mRNA expression of proliferation markers increased after treatment with AL extract (Fig. 1B); especially, *Ki-67* gene expression increased by 6.39-fold compared to the DMSO control. Keratin genes can be used as proliferation marker during keratinocyte proliferation. The expression of *KRT5/14* mRNA is increased during the division of basal proliferative keratinocytes and is reduced in upper layer keratinocytes. The expression of *KRT6/17* mRNA is increased by the hyper-proliferative signal [19]. AL extract reduced the expression of *KRT5/14* depending on the treatment concentration but significantly increased *KRT6/17* expression (*KRT6*: 502.47- % at 100 μg/mL, and *KRT17*: 124.12-% at 100 μg/mL AL extract treatment, Fig. 1C). These results indicate that AL extract promotes the proliferation of upper layer keratinocytes.

### AL extract promotes keratinocyte re-epithelialization in vitro

**Fig. 2:**
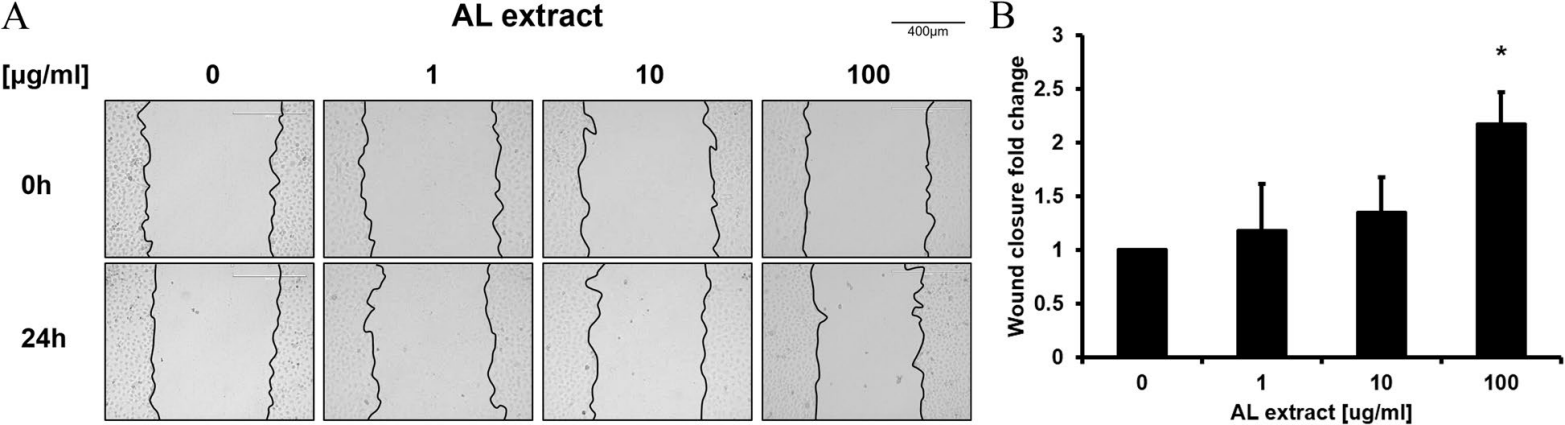
AL extract promotes keratinocyte re-epithelialization in vitro. An artificial wound was created in a monolayer of HaCaT cells and was treated with the indicated concentration of AL extract. The scratched area was compared at 0 and 24 h after AL extract treatment. **A** Representative images show the re-epithelialization. **B** Fold-change graph of (**A**). (*p < 0.05)

In vitro wound healing assays were performed to verify the re-epithelialization effect of AL extract on keratinocytes. A vertical artificial wound was formed on a HaCaT cell monolayer, and reduction of the wound area was determined by comparing the wound area at 0 h and 24 h after AL extract treatment. AL extract dose-dependently promoted wound closure by 118.1%, 135.25%, and 216.94% at 1, 10, and 100 μg/mL, respectively, compared to the DMSO control (Fig. 2). This result indicates that AL extract promotes re-epithelization of keratinocytes by activating both proliferation and migration.

### AL extract induces ERK1/2 phosphorylation

**Fig. 3:**
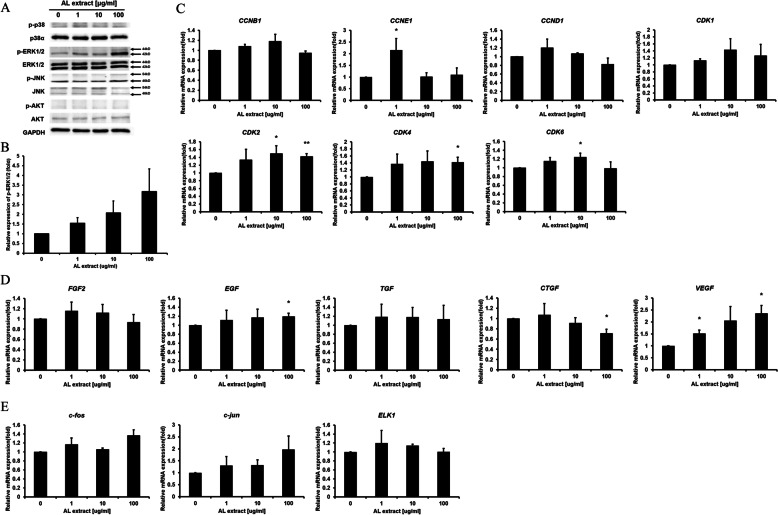
AL extract increases the expression of cell cycle regulatory genes by activating ERK1/2 in HaCaT cells. HaCaT cells were treated with the indicated concentration of AL extract. Total protein was used for Western blot analysis, and cDNA from total mRNA was used for qRT-PCR. **A** Phosphorylated forms of p38, ERK1/2, JNK, ERK5, and AKT were detected. GAPDH was used as a quantitative control. Full-length blots are presented in Supplementary Figure S1. **B** Quantity of p-ERK1/2 was calculated using densitometric analysis and normalized to GAPDH. **C** Changes in mRNA expression of gene encoding cyclins and CDKs. **D** Changes in mRNA expression of growth factors. **E** Changes in mRNA expression of AP-1 transcription factors and Elk1. (*p < 0.05, ** p < 0.01)

Western blot analysis was performed on MAP kinases (ERK1/2, JNK, p38α, and ERK5), AKT, and their respective phosphorylated forms to assess levels these intracellular regulators after AL extract treatment. Only the phosphorylated form of ERK1/2 increased with AL extract treatment (Fig. 3A and B). These results suggest that AL extract promotes keratinocyte proliferation through activation of ERK1/2.

### AL extract induces mRNA expression of proliferation regulatory genes

To verify the intracellular targets of AL extract, the mRNA expression of ERK1/2 regulatory cell proliferation genes was assessed using qRT-PCR. Cyclins and Cyclin-dependent kinases (CDK) are proteins that regulate the progression of the cell cycle. The mRNA expression of the genes encoding Cyclin B1 and CDK1/2/4/6 was increased by AL extract treatment (Fig. 3C). Growth factors stimulate cell growth by activating various signaling pathways, including the ERK1/2 kinase pathway. mRNA expression of the vascular endothelial growth factor (*VEGF*) was increased in a dose-dependent manner (235.8% at 100 μg/mL), while the expression of the connective tissue growth factor (*CTGF*) was reduced (71.4% at 100 μg/mL). The mRNA expression of other growth factors did not show significant changes with AL extract treatment (Fig. 3D). The AP-1 transcription factors (c-Jun and c-Fos) and Elk1 regulate the transcription of genes that code for cell cycle regulatory proteins. mRNA expression of the gene encoding Elk1 was unchanged, but the expression of *c-jun* and *c-fos* increased with AL extract treatment (Fig. 3E). These results indicate that AL extract promotes the proliferation of keratinocytes by inducing the mRNA expression of *c-fos*, *c-jun*, and *VEGF*.

### AL extract promotes wound healing in vivo

**Fig. 4:**
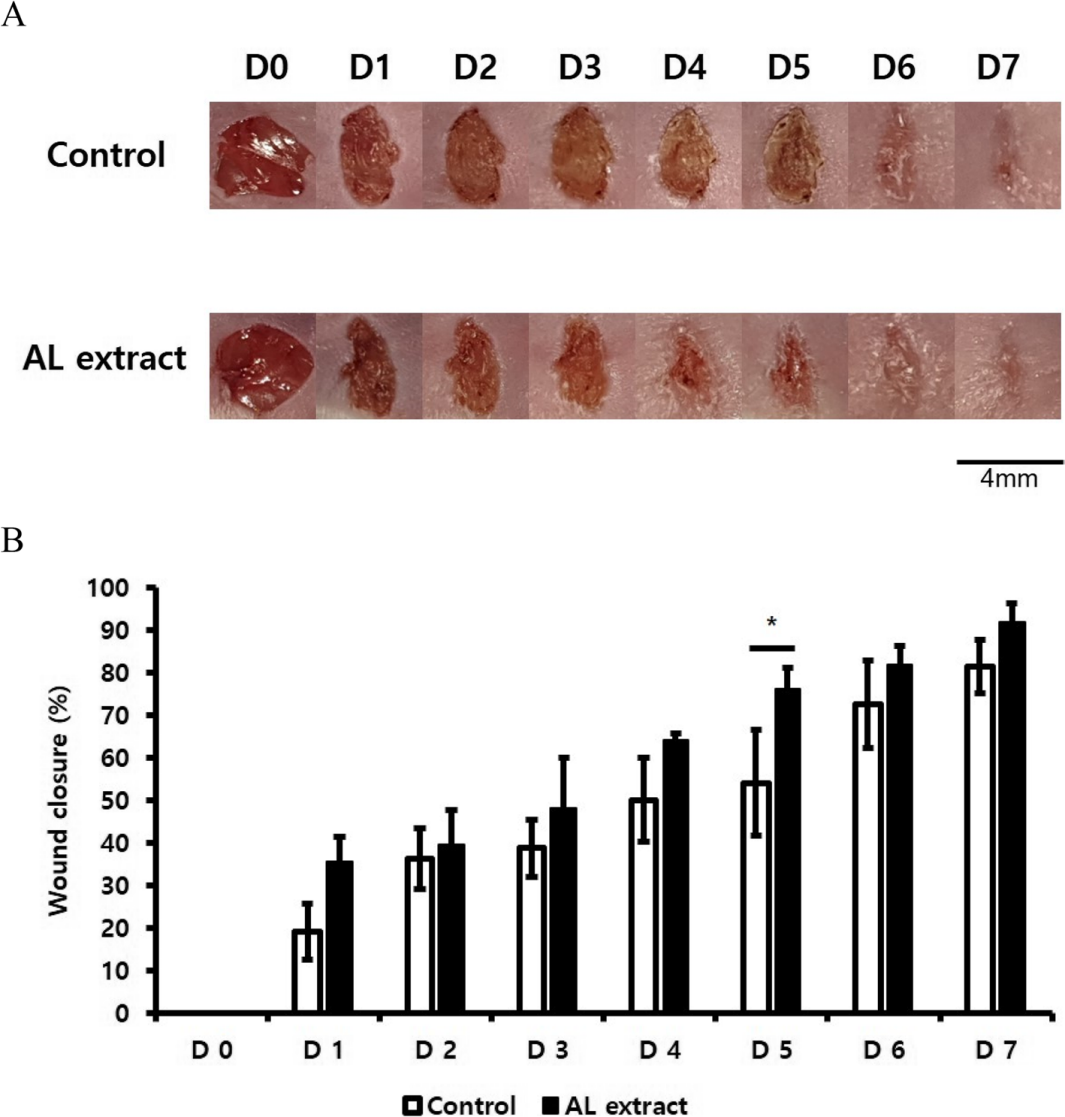
AL extract promotes wound-healing in vivo. Wounds (4 mm diameter) were created on the backs of six- to seven-week-old BALB/c mice. AL extract was administered at a dose of 50 μg/day. **A** Daily images show the difference in wound healing progression in vivo. **B** Graph shows the percentage of wound closure in (**A**). (*p < 0.05)

In vivo wound healing assays were conducted to verify the wound-healing efficacy of AL extract under in vivo conditions. Wound healing activity was improved in the AL extract-treated wound area compared to the DMSO control (Fig. 4). The greatest difference in wound closure was observed on the 5th day (22.13%).

## Discussion

In this study, we have demonstrated the wound-healing potential of AL extract. AL extract treatment induced mRNA expression of proliferation markers and *KRT6/17*, which are expressed in hyper-proliferative signals [19] (Fig. 1C). Conversely, the mRNA expression of *KRT5/14* was decreased by AL extract treatment (Fig. 1C). *KRT5/14* is typically expressed in proliferating basal layer keratinocytes and their expression is down-regulated during differentiation [20]. This result suggests that in addition to promoting proliferation, AL extract also promotes the differentiation of keratinocytes. The proliferation-promoting effect of AL extract induced re-epithelialization under in vitro conditions (Fig. 2), and its efficacy in promoting actual biological wound healing was confirmed by the in vivo wound healing assay (Fig. 4). These effects were attributed to the activation of cell signaling pathway.

AL extract treatment induced the phosphorylation of MAP kinase ERK1/2 (Fig. 3A), resulting in a significant increase in mRNA expression of *c-jun*, *c-fos*, and *VEGF* (Fig. 3D and E). Activation of the ERK1/2 signaling pathway in proliferative conditions triggers the expression of AP-1 transcription factors and cyclins [5, 21]. AP-1 transcription factors, particularly c-Jun and c-Fos, play a crucial role in cell cycle progression in proliferating cells by controlling the expression of various cell cycle regulatory genes, including cyclins, CDKs, and VEGF [22–24]. These findings suggest that AL extract leads to processes such as wound healing, angiogenesis, endothelial cell migration, and vascular permeability by activating the ERK1/2 pathway in keratinocytes [25, 26].

Our previous studies on wound healing consistently demonstrated phosphorylation of ERK1/2. In recent studies, *Trichosanthes kirilowii* Maxim extract showed that phosphorylation of ERK1/2 promotes the proliferation of keratinocytes by increasing the mRNA expression of *c-fos*, *c-jun*, *VEGF*, and *CTGF* [27, 28]. However, interestingly, the mRNA expression of *CTGF*, which is also activated by the ERK1/2 pathway, and c-Jun and c-Fos [29] was dose-dependently decreased by treatment with AL extract. This result suggests that AL extract treatment reduces *CTGF* mRNA expression through other signaling pathways or proteins. Differences in mechanism can be attributed to variation in the composition of constituents.

AL extract contains various bioactive compounds such as steroidal saponins, phenolic compounds, flavonoids, alkaloids, and polysaccharides [30–32]. However, the specific key compounds responsible for its wound healing effects have not been identified in this study. Conducting comparative analyses of the major compounds in AL extract could provide valuable insights into their roles and potential synergistic effects. This information is essential for the development of novel wound healing agents and advancements in pharmaceutical research. In our further study, we will focus on examining the wound healing efficacy of single compound present in plant extract and studying the mechanism by which they regulate selective gene expression after signal pathway activation.

## Conclusion

In this study, we found that AL extract promotes wound healing both in vitro and in vivo by enhancing the proliferation of keratinocytes. These effects were mediated through increased expression of *c-jun*, *c-fos*, and *VEGF*, which were induced by phosphorylation of ERK1/2. Based on the results of this study, it is suggested that AL extract has potential as a natural and effective therapeutic agent for skin regeneration and wound healing applications, although further research is needed to fully elucidate the underlying mechanisms.

## Methods

### Plant material and extraction

*Asparagus lucidus* Lindl (Cheonji Gayakcho, South Korea) extraction was performed as previously reported [28, 30]. Freeze dryer (IlShin, Dongducheon, Korea), rotary evaporator (EYELA, Tokyo, Japan), and refrigerated bath circulator (JEIO TECH, Daejeon, Korea) were used in this study. Using 500 g of *Asparagus lucidus* Lindl, a total of 65.7 g lyophilized AL extract powder was formed (yield: 13.14%). The extract was dissolved in DMSO (10 mg/mL) and stored at 4 ℃ before use.

### Cell culture and AL extract treatment

The human keratinocyte cell line HaCaT (kindly provided by COSMAX BIO, Jecheon, Korea) was incubated in Dulbecco’s Modified Eagle’s Medium (DMEM) containing 10% fetal bovine serum and 1% penicillin–streptomycin at 37 °C and 5% CO_2_. HaCaT cells were seeded at 10^3^ cells per well in 96-well plates and 3 × 10^5^ cells per well in 6-well plates. After 24 h, DMEM medium was replaced with a serum-free medium containing the indicated concentration of AL extract, and cells were incubated for an additional 24 h [27, 28].

### MTT assay

MTT assays were performed as previously reported [27, 28]. MTT (3-(4,5-dimethyl-thiazol-2yl)-2,5-diphenyltetrazolium bromide, Sigma Aldrich, St. Louis, MO, USA) and a microplate reader (BioTek Instruments, Winwooski, VT, USA) were used in this study. The experiments were independently repeated three times.

### In vitro wound healing assay

In vitro wound healing assays were performed as previously reported [28]. A 0.1–10 µL white tip (Sorenson, Salt Lake City, UT, USA), and EVOS XL imaging system (Fisher Scientific, Hampton, NH, USA) were used in this study. The wound closure area was calculated as follows:

$$\mathrm{Wound}\;\mathrm{closure}\;\mathrm{area}\;\left(\mathrm\mu\mathrm m^2\right)=0\;h\;\mathrm{wound}\;\mathrm{area}-24\;h\;\mathrm{wound}\;\mathrm{area}$$

The experiments were independently repeated three times.

### Western blot assays

Western blots were performed as previously reported [27, 28]. Antibodies for p38 (sc-535), JNK1/2 (sc-7345), p-JNK1/2 (sc-6254), ERK5 (sc-398015), p-ERK5 (sc-135760), GAPDH (sc-25778), and goat anti-rabbit IgG-HRP secondary antibody were purchased from Santa Cruz Biotechnology (Dallas, TX, USA). Antibodies for p-p38 (#9211), ERK1/2 (#9102), p-ERK1/2 (#9101), AKT (#9272), and p-AKT (#9271) were purchased form Cell Signaling Technology (Danvers, MA, USA), and goat anti-mouse IgG-HRP secondary antibody was purchased from Bio-Rad (Hercules, CA, USA). The experiments were independently repeated three times.

### Quantitative real-time PCR

Quantitative real-time PCR was performed as previously reported [28, 33]. The TRIzol reagent (Fisher Scientific, Hampton, NH, USA), Thermo reverse transcriptase (NanoHelix, Daejeon, Korea), and QGreen 2 × SybrGreen qPCR Master Mix (CellSafe, Yongin, Korea) were used in this study. A total of 1 µg RNA was used for cDNA synthesis. mRNA expression change was calculated using the ΔΔCt formula. The gene encoding GAPDH was used as the quantitative control. Primer sequences used in this study are listed in Table 1. The experiments were independently repeated three times.

**Table 1:** Primer sequences used in this study

Genes	Forward	Reverse	Reference
*PCNA*	AACCTCACCAGTATGTCCAA	ACTTTCTCCTGGTTTGGTG	[34]
*Ki-67*	CCAAAGAAGGCTGAGGACAA	CCCTTAAGCAGACTGACAGC	[28]
*MCM2*	AATCTATGGCGACAGGCAG	ATCACATAGTCCCGCAGAT	[28]
*CCND1*	CTGTGCTGCGAAGTGGAAACC	GACGATCTTCCGCATGGAC	[28]
*CCNB1*	TAAGGCGAAGATCAACATGG	GCTTCCTTCTTCATAGGCAT	[28]
*CCNE1*	ACACCATGAAGGAGGACG	CACAGACTGCATTATTGTCCC	[28]
*CDK1*	CAGGTCAAGTGGTAGCCATG	ACCTGGAATCCTGCATAAGC	[28]
*CDK2*	TTCTCATCGGGTCCTCCACC	TCGGTACCACAGGGTCACCA	[28]
*CDK4*	CTGAGAATGGCTACCTCTCG	CGAACTGTGCTGATGGGAAG	[28]
*CDK6*	CCGAAGTCTTGCTCCAGTCC	GGGAGTCCAATCACGTCCAA	[28]
*KRT5*	AGCAGTGGTACGCTTGTTGATT	GCCTGGACTCAGAGCTGAGAA	[35]
*KRT14*	GGCCTGCTGAGATCAAAGACTAC	CACTGTGGCTGTGAGAATCTTGTT	[36]
*KRT6*	CTGAGGCTGAGTCCTGGTAC	GTTCTTGGCATCCTTGAGG	[28]
*KRT17*	GCTGCTACAGCTTTGGCTCT	TCACCTCCAGCTCAGTGTTG	[37]
*c-fos*	GGAGGAGGGAGCTGACTGATA	GCAATCTCGGTCTGCAA	[38]
*c-jun*	TTCTATGACGATGCCCTCAACGC	GCTCTGTTTCAGGATCTTGGGGTTAC	[38]
*ELK1*	CTGACCCCATCCCTGCTTCCTA	GAAGTGAATGCTAGGAGGCAGCG	[38]
*FGF2*	AAAAACGGGGGCTTCTTCCT	AGCCAGGTAACGGTTAGCAC	[39]
*EGF*	AGTCCGTGACTTGCAAGAGG	CCTCTTCTTCCCTAGCCCCT	[39]
*TGF*	TGGTGGAAACCCACAACGAA	GAGCAACACGGGTTCAGGTA	[39]
*CTGF*	GTTTGGCCCAGACCCAACTA	GGCTCTGCTTCTCTAGCCTG	[39]
*VEGF*	CTTGCCTTGCTGCTCTACCT	GCAGTAGCTGCGCTGATAGA	[39]
*GAPDH*	GTATCGTGGAAGGACTCATG	GAGGCAGGGATGATGTTC	[28]

### In vivo wound healing assay

In vivo wound healing assays were performed previously reported [28, 40, 41]. Six- to seven-week-old female BALB/c AnNTac mice (JA BIO, Korea) were used. The mice were housed under a 12-h light–dark cycle and provided with optimal temperature and humidity conditions. Cage cleaning was conducted every 3–4 days, based on the level of contamination. Avertin (2,2,2-Tribromoethanol, Sigma, USA) at 125 mg/kg was used to anesthetize the mice by intraperitoneal injection. Mouse back hair was completely removed using animal clipper (JEUNG DO BIO & PLANT CO, Korea) and Hair Removal Cream (BEAUTY FORMULAS, Korea). Three days after hair removal, a 4 mm-diameter biopsy punch (KEYES, Germany) was used to form full-thickness wounds below both shoulder blades. To prevent interference from other individuals, each cage housed one mouse after wound creation. The left-side wound was treated with 10% DMSO and the right-side wound treated with 50 μg (5 μg/μl in 10% DMSO) of AL extract daily. After applying the treatments to the respective wound sites, sufficient time was given for absorption. The wound area was photographed every 24 h. Three animals were used as one experimental group. After the completion of the experiment, the experimental animals were euthanized in accordance with ethical guidelines. This study was conducted with the approval of the Animal Experimental Ethics Committee of Kyung Hee University (approval number: KHGASP-20–560) and performed under the Institutional Animal Care and Use Committee guidelines. The experiments were independently repeated three times.

$$\mathrm{Wound}\;\mathrm{closure}\;\left(\%\right)=\frac{\mathrm D0\;\mathrm{wound}\;\mathrm{area}-\mathrm Dx\;\mathrm{wound}\;\mathrm{area}}{\mathrm D0\;\mathrm{wound}\;\mathrm{area}}\times100$$

### Statistical analysis

The experiments were independently repeated three times. Values are expressed as mean ± standard deviation. One-way ANOVA was used in analyzing statistically significant differences. Two group comparisons were analyzed using the Student’s t-test. (*p < 0.05, **p < 0.01, ***p < 0.001).

## Supplementary Information

**Additional file 1.** 

## Data Availability

All data generated or analyzed during this study are included in this published article.
