# Supplementary material for: Wound healing effects of Asparagus lucidus Lindl extract through the phosphorylation of ERK1/2
Source: BMC Complement Med Ther. 2023 Jul 15;23:238. doi: 10.1186/s12906-023-04066-w (PMC10349518; doi:10.1186/s12906-023-04066-w)

**Supplementary material**

**Figure S1.** Original images of all blots in Figure 3A.

(A) Original images of all blots of p-p38 (Including all repeated images).


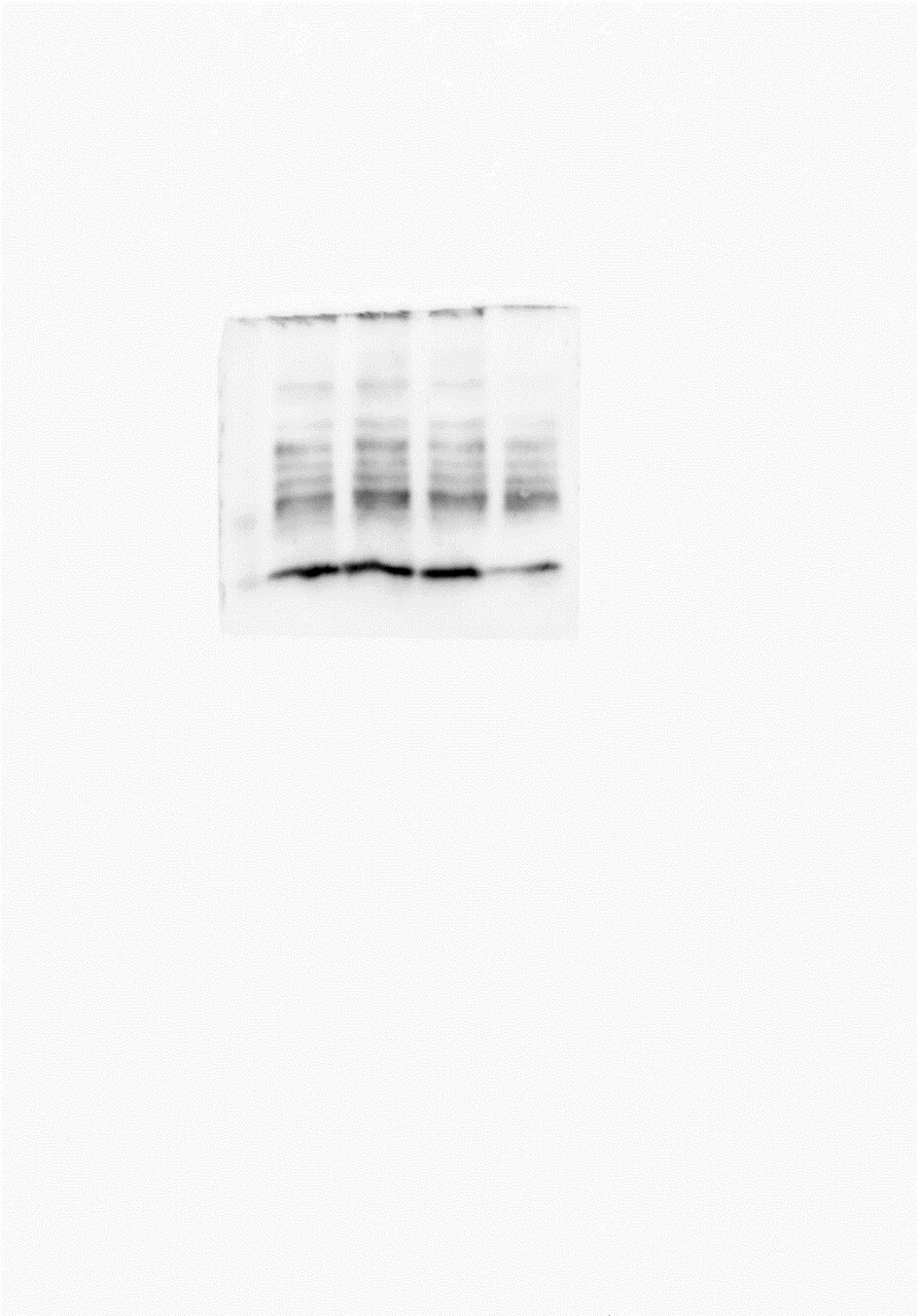

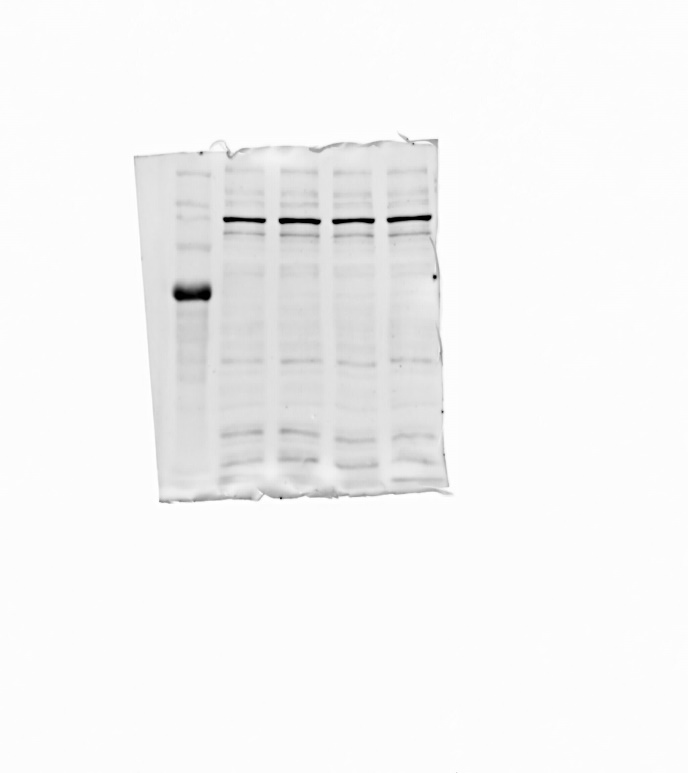

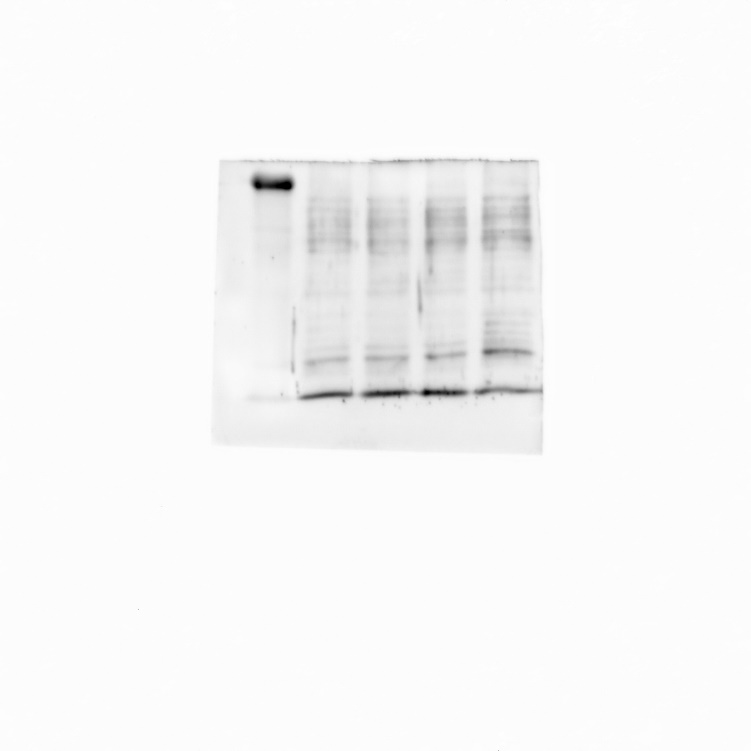


(B) Original images of all blots of p38 (Including all repeated images).


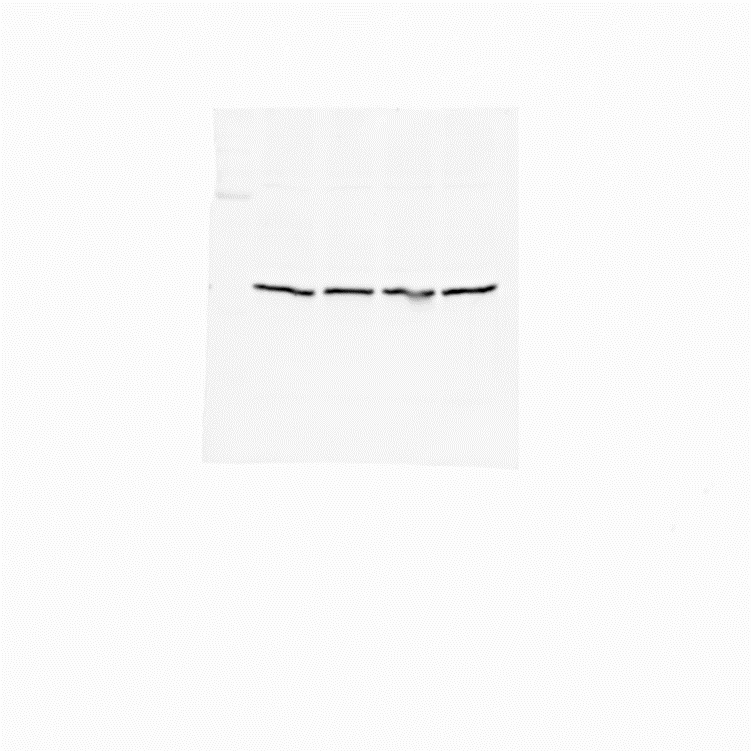

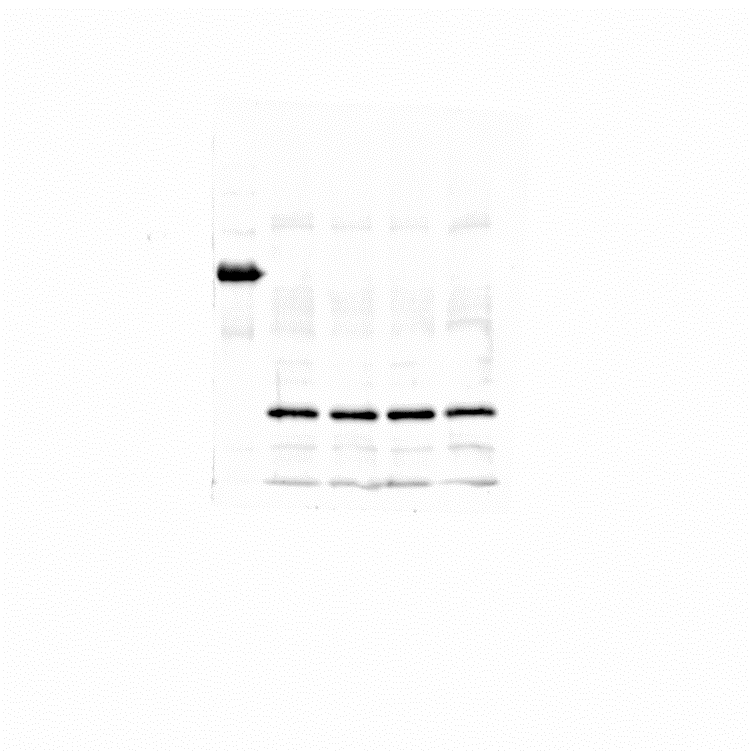

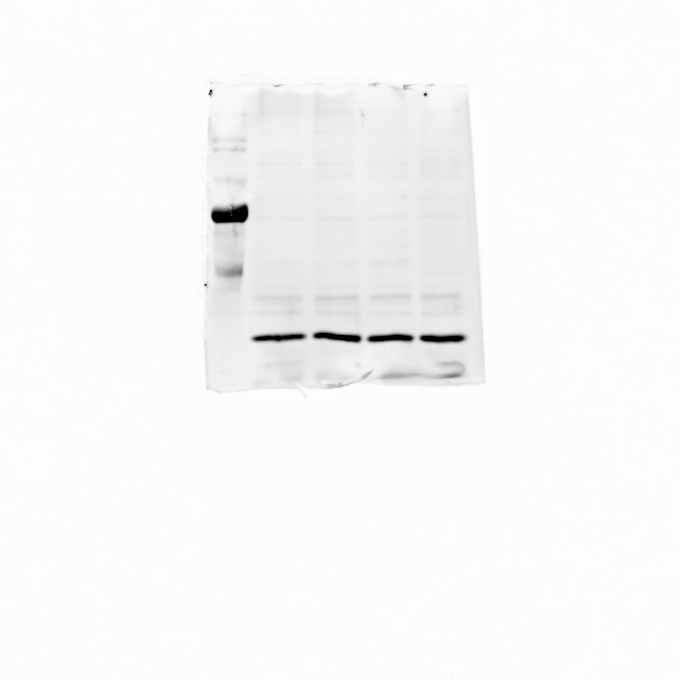


(C) Original images of all blots of p-ERK1/2 (Including all repeated images).


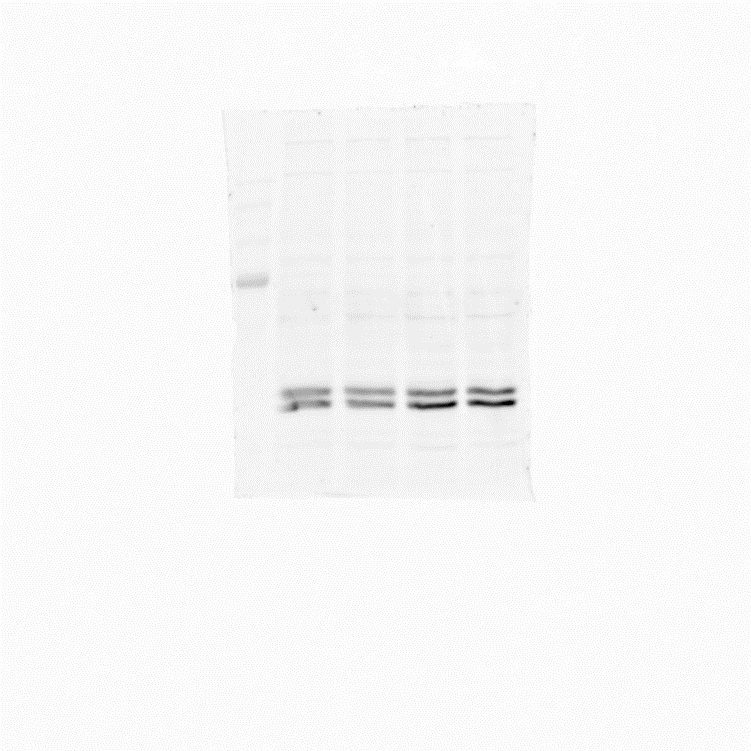

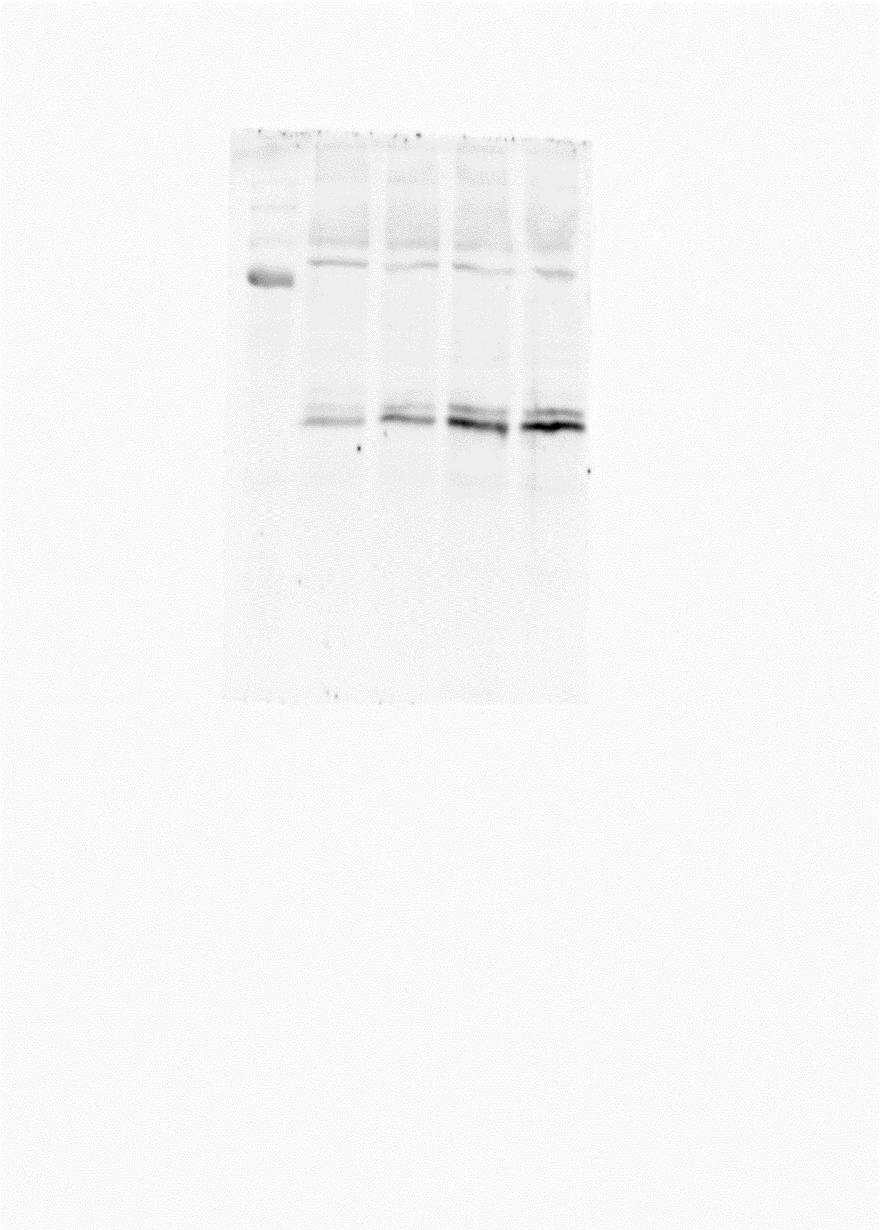

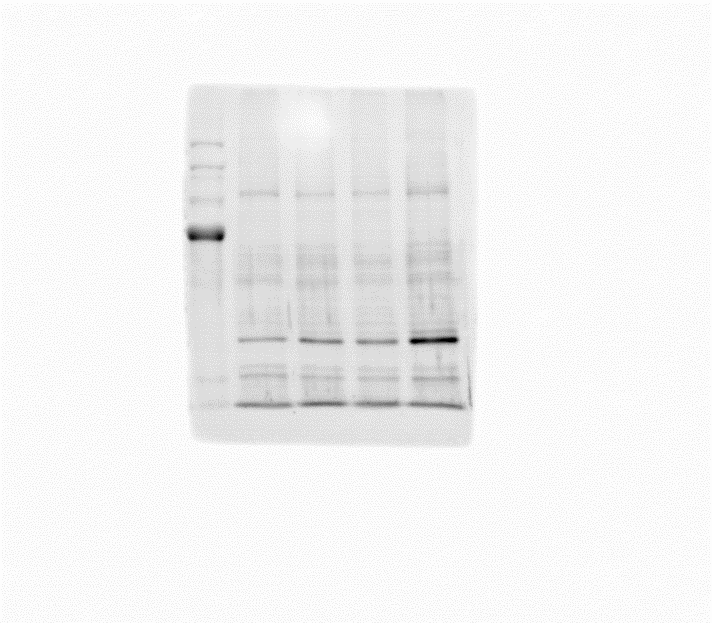


(D) Original images of all blots of ERK1/2 (Including all repeated images).


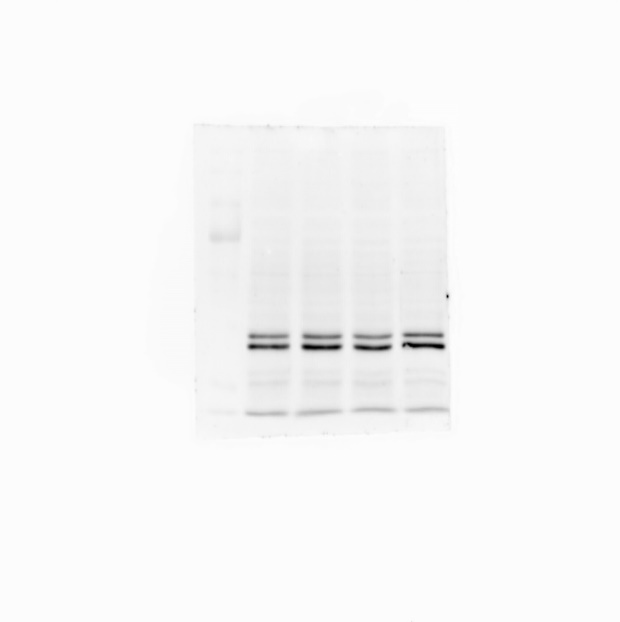

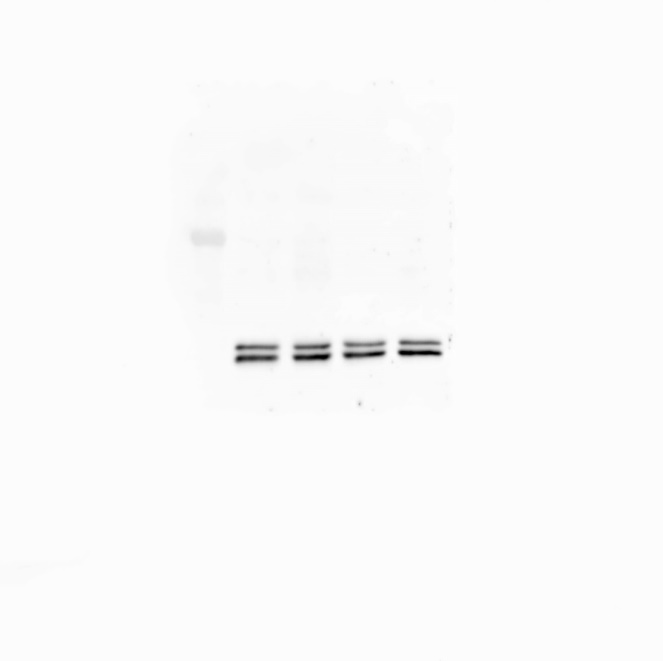

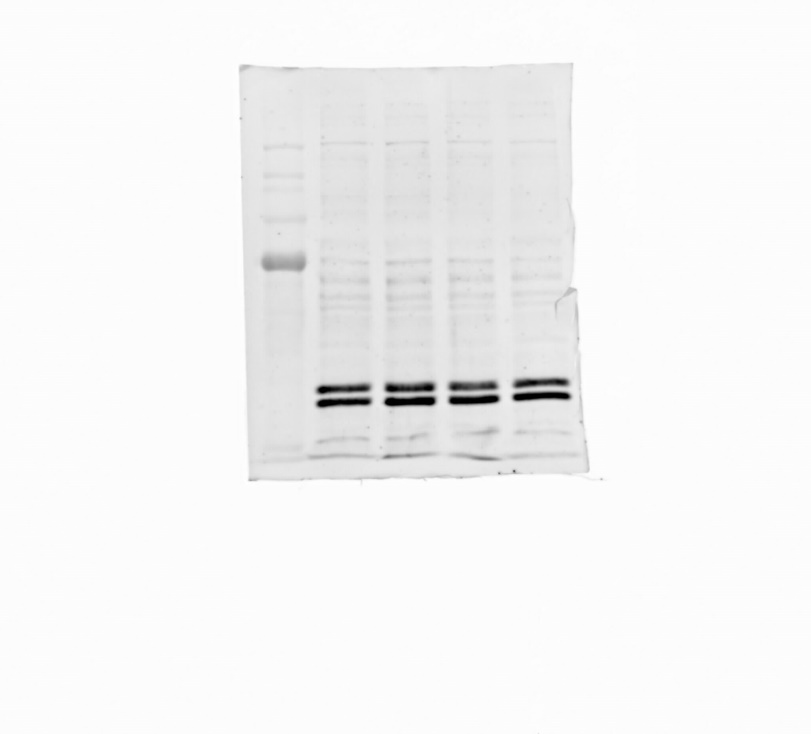

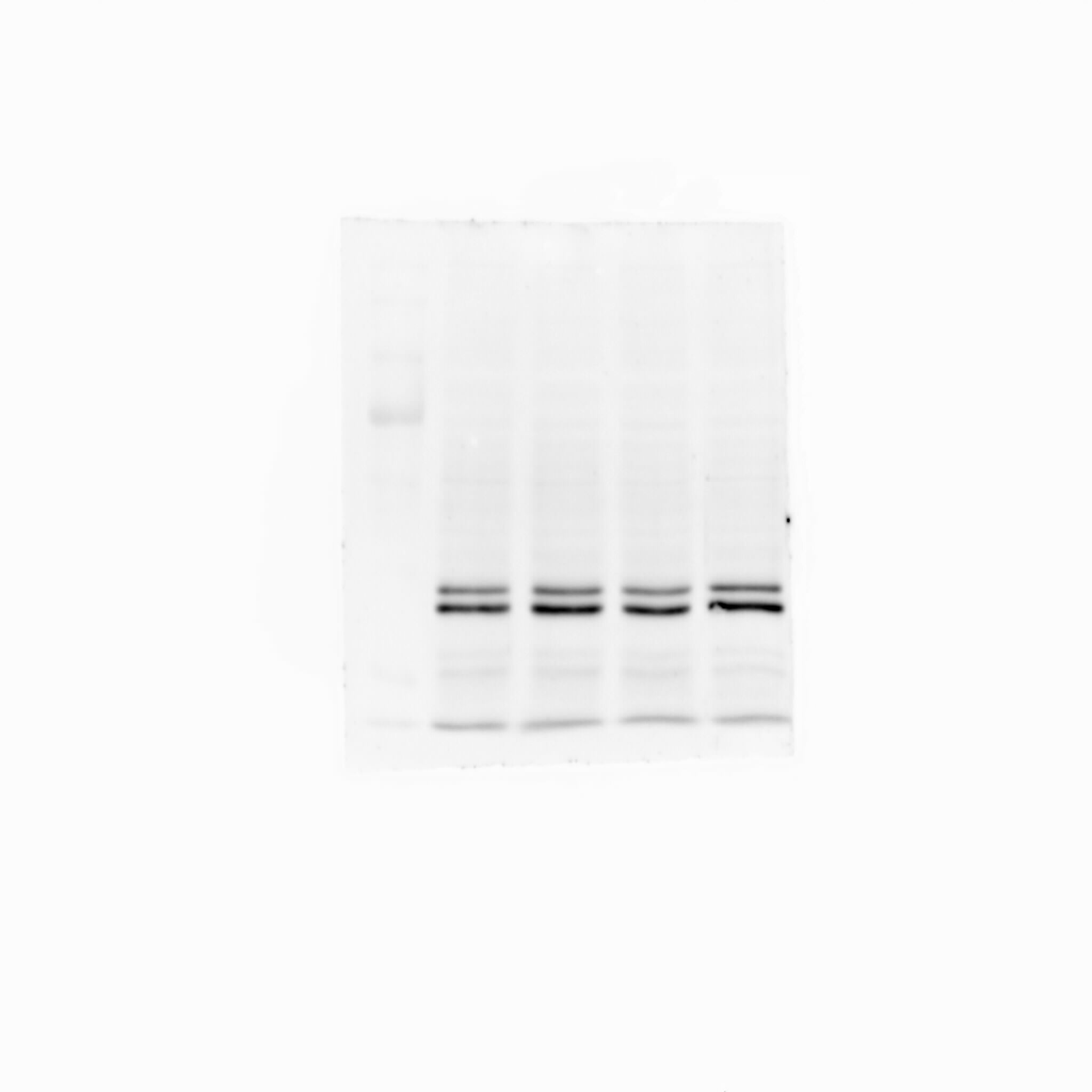


(E) Original images of all blots of p-JNK (Including all repeated images).


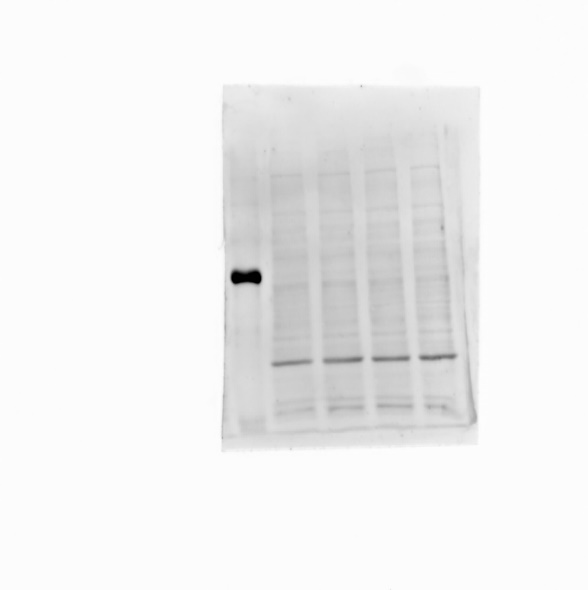

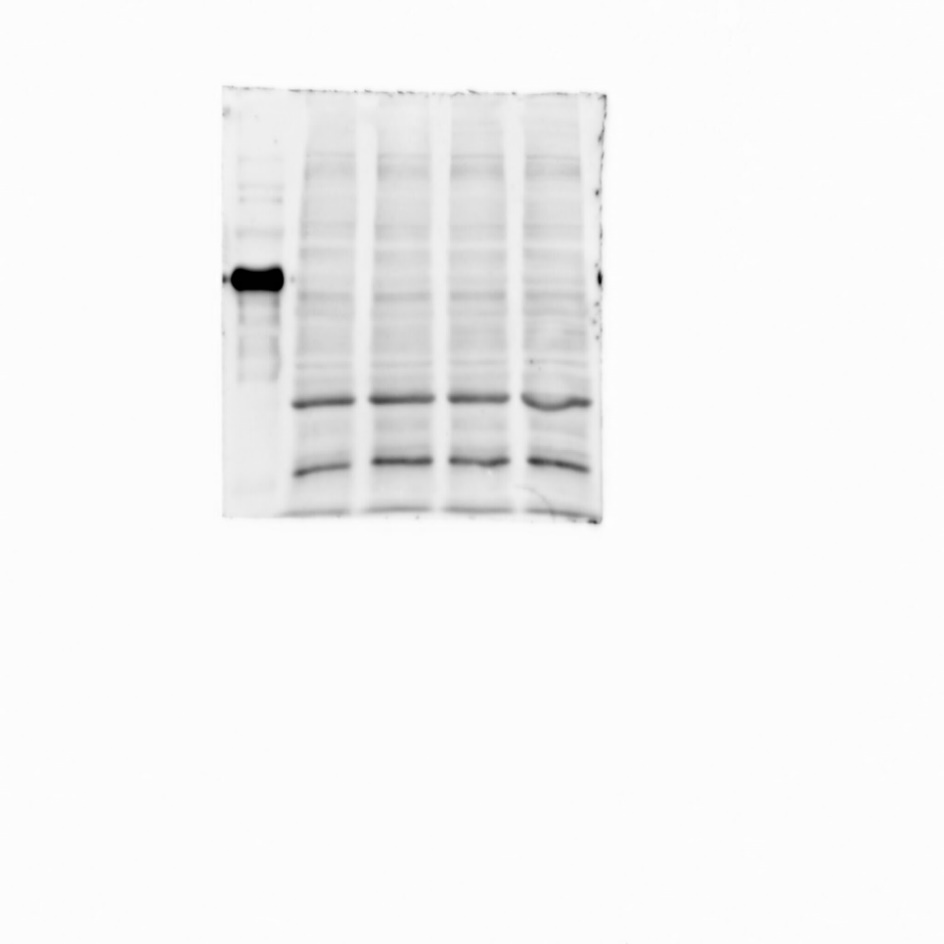

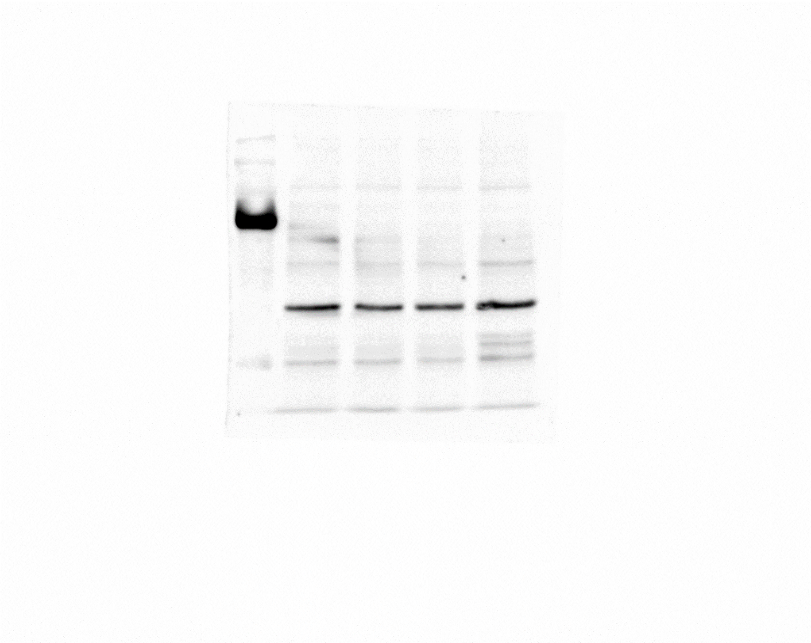


(F) Original images of all blots of JNK (Including all repeated images).


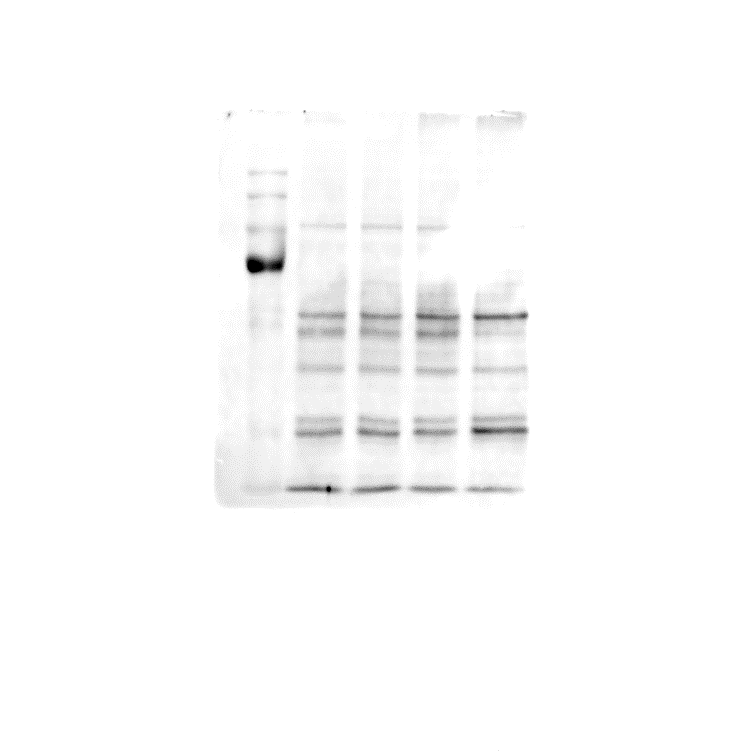

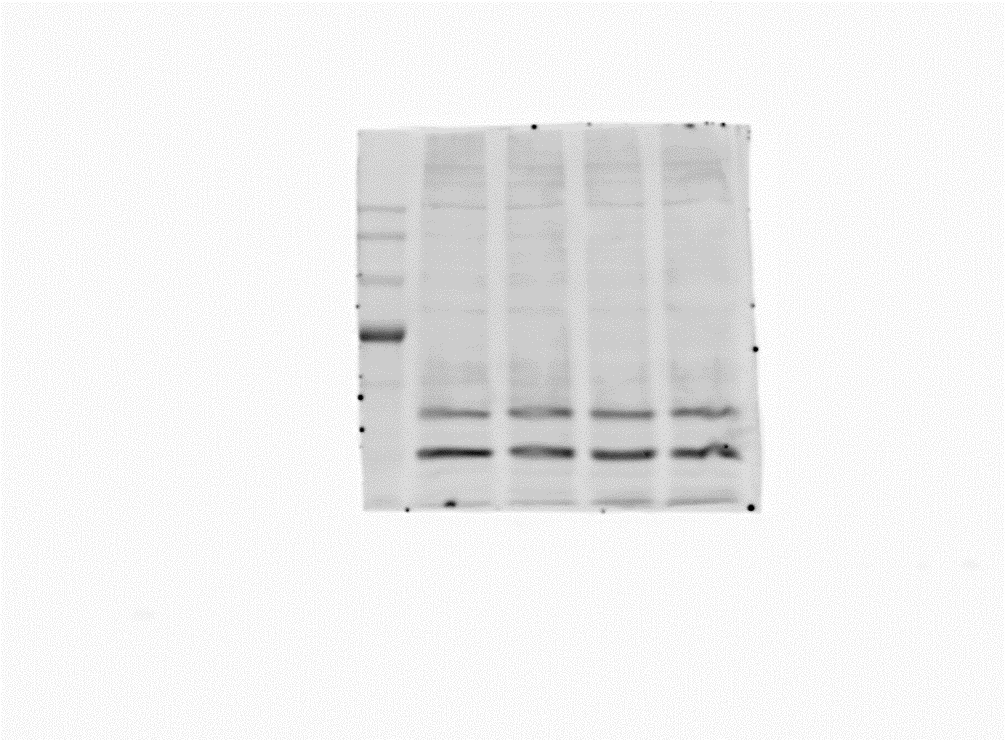

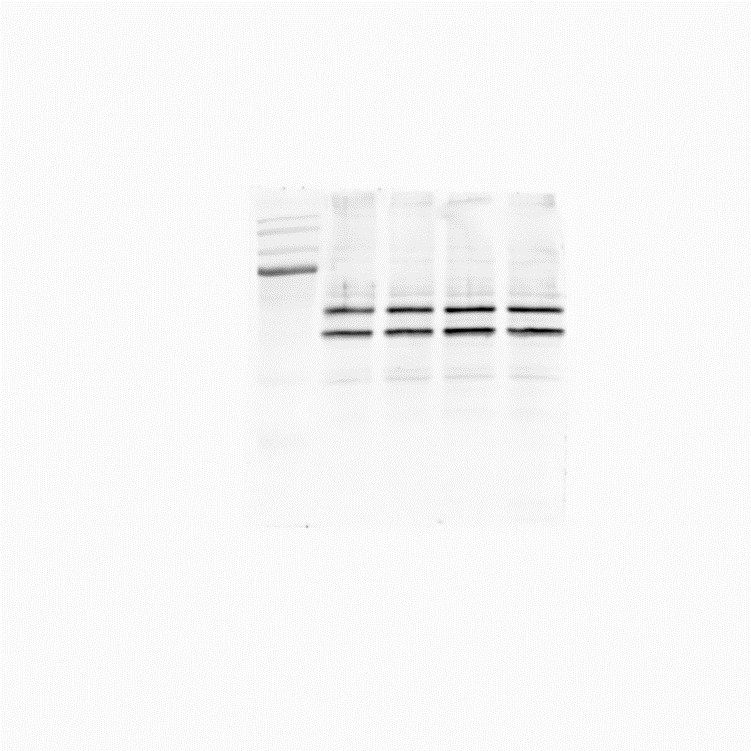


(G) Original images of all blots of p-AKT (Including all repeated images).


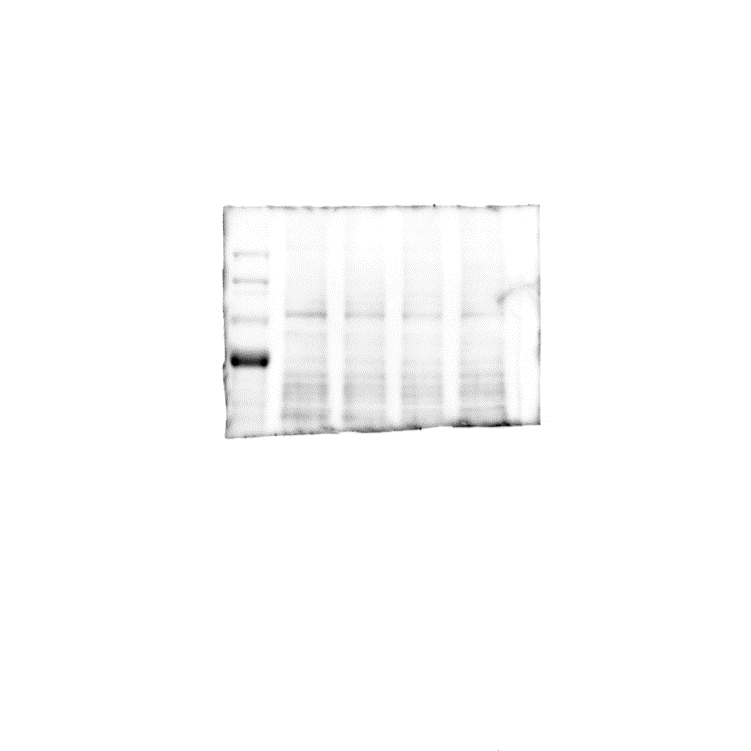

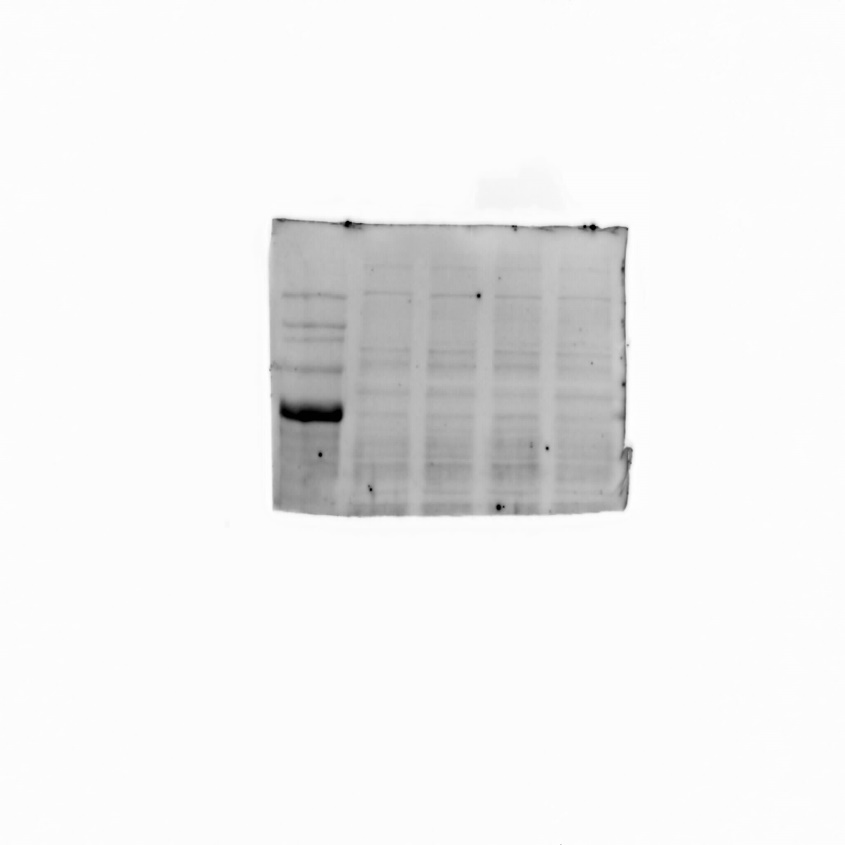

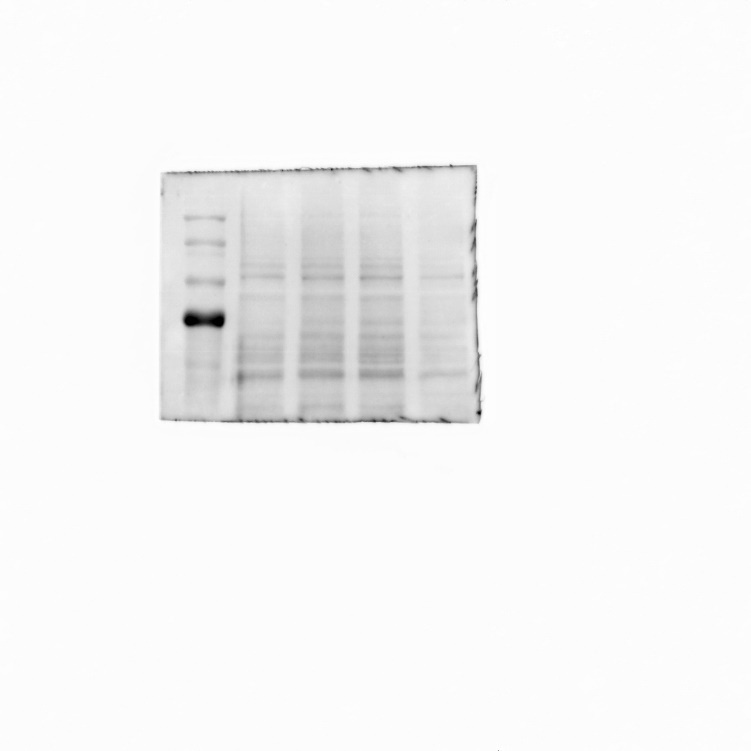


(H) Original images of all blots of AKT (Including all repeated images).


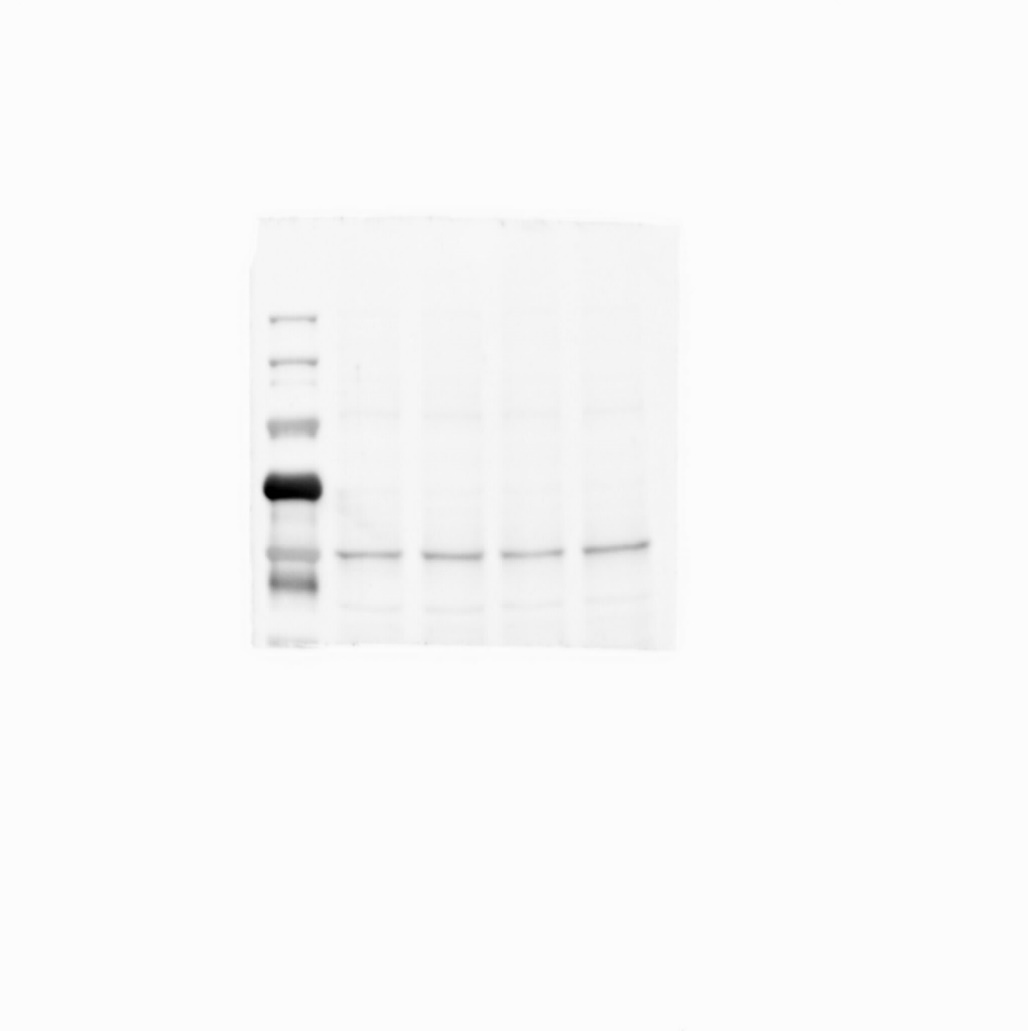

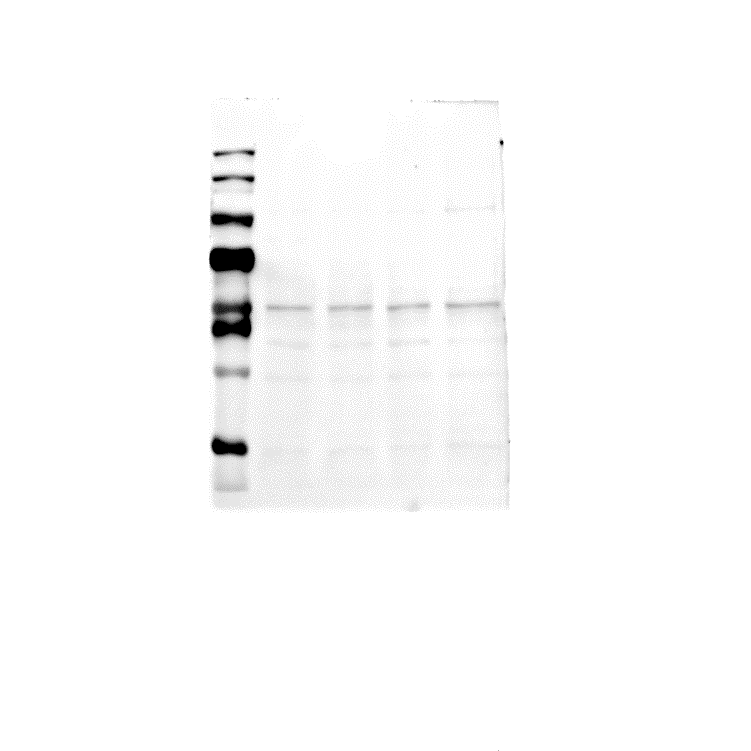

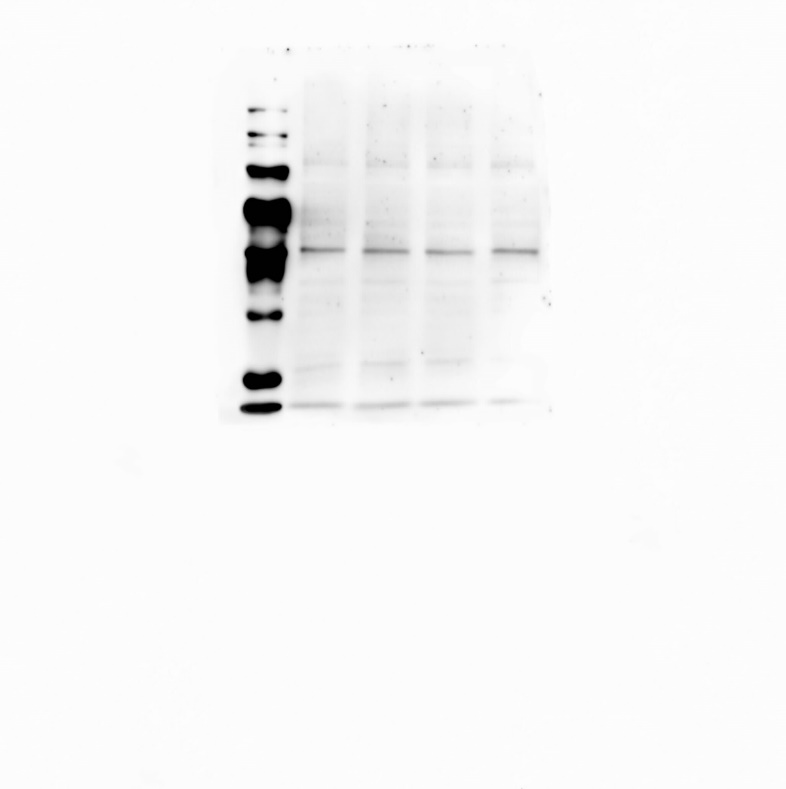


(I) Original images of all blots of GAPDH (Including all repeated images).


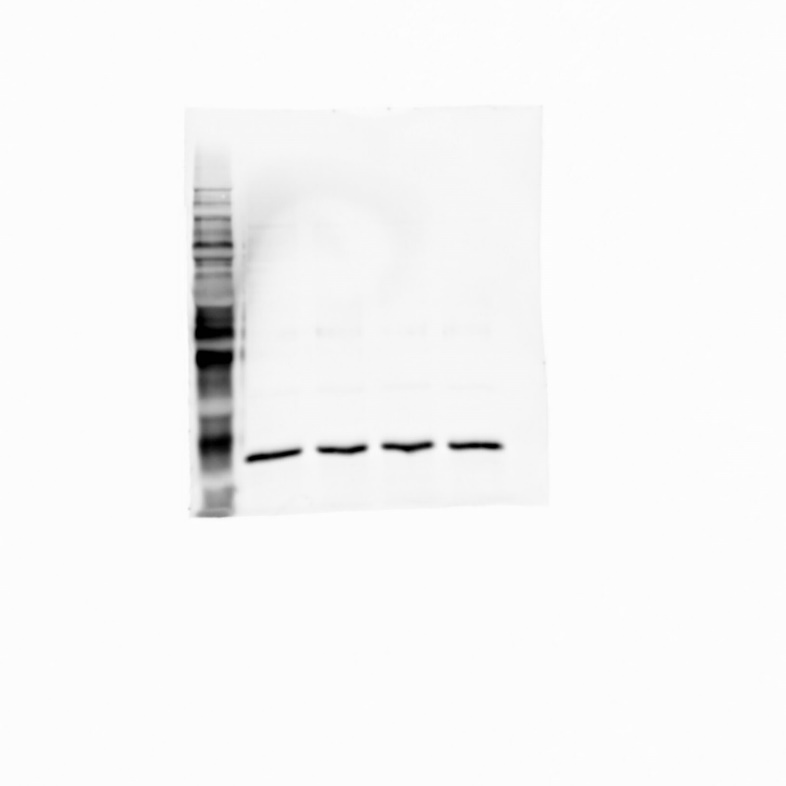

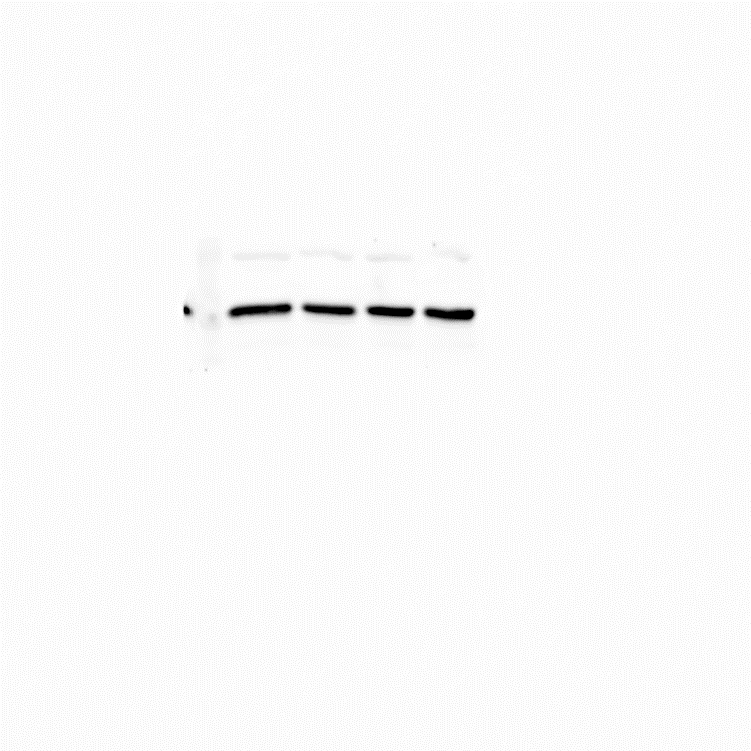

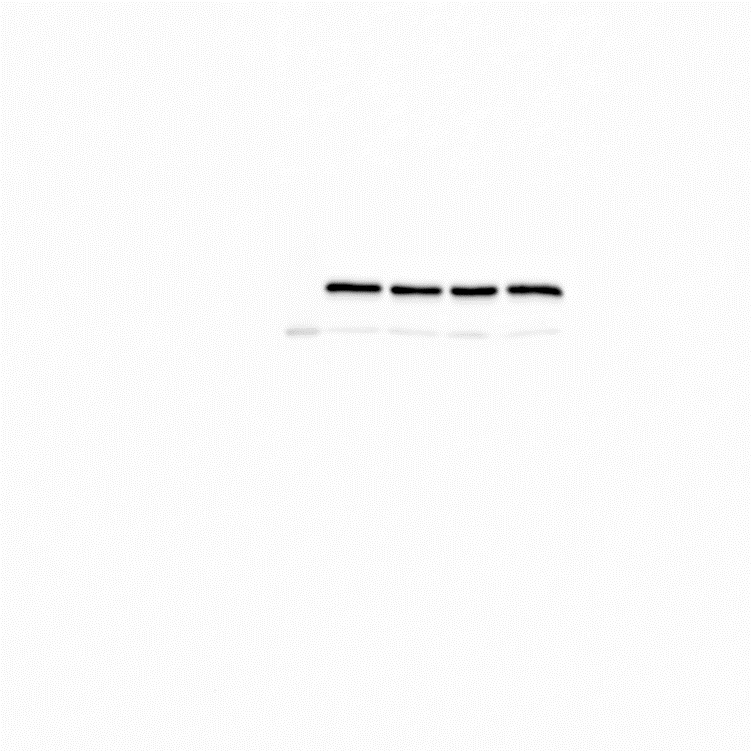

Supplement: Supplementary file 1 — Additional file 1. [file 12906_2023_4066_MOESM1_ESM.docx]
